# Fluorescence resonance energy transfer sensors for quantitative monitoring of pentose and disaccharide accumulation in bacteria

**DOI:** 10.1186/1754-6834-1-11

**Published:** 2008-06-03

**Authors:** Thijs Kaper, Ida Lager, Loren L Looger, Diane Chermak, Wolf B Frommer

**Affiliations:** 1 Department of Plant Biology, Carnegie Institution of Washington, Panama Street, Stanford, CA 94305, USA; 2 Danisco US Inc., Genencor Division, Page Mill Road, Palo Alto, CA 94304, USA; 3 Department of Cell and Organism Biology, Lund University, Sölvegatan 35, 223 62 Lund, Sweden; 4 Janelia Farm, Howard Hughes Medical Institute, Helix Drive, Ashburn, VA 20147, USA

## Abstract

**Background:**

Engineering microorganisms to improve metabolite flux requires detailed knowledge of the concentrations and flux rates of metabolites and metabolic intermediates *in vivo*. Fluorescence resonance energy transfer sensors represent a promising technology for measuring metabolite levels and corresponding rate changes in live cells. These sensors have been applied successfully in mammalian and plant cells but potentially could also be used to monitor steady-state levels of metabolites in microorganisms using fluorimetric assays. Sensors for hexose and pentose carbohydrates could help in the development of fermentative microorganisms, for example, for biofuels applications. Arabinose is one of the carbohydrates to be monitored during biofuels production from lignocellulose, while maltose is an important degradation product of starch that is relevant for starch-derived biofuels production.

**Results:**

An *Escherichia coli *expression vector compatible with phage λ recombination technology was constructed to facilitate sensor construction and was used to generate a novel fluorescence resonance energy transfer sensor for arabinose. In parallel, a strategy for improving the sensor signal was applied to construct an improved maltose sensor. Both sensors were expressed in the cytosol of *E. coli *and sugar accumulation was monitored using a simple fluorimetric assay of *E. coli *cultures in microtiter plates. In the case of both nanosensors, the addition of the respective ligand led to concentration-dependent fluorescence resonance energy transfer responses allowing quantitative analysis of the intracellular sugar levels at given extracellular supply levels as well as accumulation rates.

**Conclusion:**

The nanosensor destination vector combined with the optimization strategy for sensor responses should help to accelerate the development of metabolite sensors. The new carbohydrate fluorescence resonance energy transfer sensors can be used for *in vivo *monitoring of sugar levels in prokaryotes, demonstrating the potential of such sensors as reporter tools in the development of metabolically engineered microbial strains or for real-time monitoring of intracellular metabolite during fermentation.

## Background

Recent economic and geopolitical factors have instigated efforts for the economically competitive conversion of biomass-derived carbohydrates to combustibles that can replace petroleum-based liquid fuels. Ethanol is used as a renewable transportation fuel replacing an increasing part of automotive fuel. In the US, ethanol is currently produced mainly by yeast-mediated fermentation of glucose derived from corn starch; however, the energy balance is not optimal [1]. Lignocellulosic biomass is an alternative feedstock that can be fermented to ethanol or other biofuels after appropriate pretreatment. Lignocellulosic biofuels are expected to have significantly higher energy efficiency [2]. Efficient utilization of lignocellulose will require engineering of the feedstock, deconstruction as well as fermentation [3]. For example, the lignocellulose of corn fiber, in contrast to corn starch, contains hexoses as well as a variety of pentoses, such as xylose and arabinose, which are derived from hemicellulose and that are not efficiently fermented by the yeast *Saccharomyces cerevisiae*. Selection of suitable strains combined with genetic engineering of *S. cerevisiae, Escherichia coli *and *Zymomonas mobilis *are used to improve pentose utilization [4-7].

**Figure 1:**
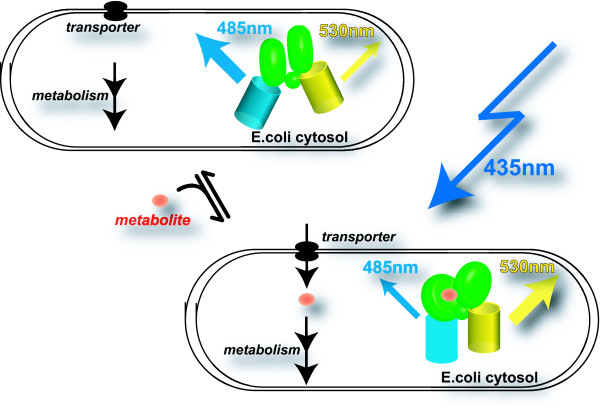
***In vivo *analysis of metabolite accumulation in live bacterial cells using fluorescence resonance energy transfer sensors**. Gram-negative bacterial cells are shown surrounded by two membranes expressing the fluorescence resonance energy transfer sensor, which consists of an episomally encoded recognition element (green central domain composed of two lobes that both contribute to ligand binding, connected by a hinge region), here a periplasmic binding protein for arabinose or maltose, sandwiched by an N-terminal cyan variant of green fluorescent protein (enhanced cyan fluorescent protein) and a C-terminal yellow variant (enhanced yellow fluorescent protein). The sensor contains no targeting information and will thus be present in the cytosol of *Escherichia coli*. Excitation of enhanced cyan fluorescent protein with 435 nm light results in emission from enhanced cyan fluorescent protein (peak emission at ~485 nm) and fluorescence resonance energy transfer-derived enhanced yellow fluorescent protein emission (peak emission at ~528 nm). The important characteristics of the sensor are the binding constant and detection range. The cell in the top panel is cultivated in the absence of the ligand; therefore, endogenous ligand concentrations are low (all sensors are in the unbound state, with low fluorescence resonance energy transfer). The cell in the bottom panel is cultivated in the presence of the ligand; therefore, endogenous ligand concentrations are high (all sensors are in the bound state, with high fluorescence resonance energy transfer). The steady-state levels and accumulation rates in the cytosol will be determined predominantly by the external ligand levels as well as the properties and abundance of transporters and the downstream metabolic enzymes.

Fluxome analysis is used to determine the bottlenecks in metabolic pathways that limit fermentative capacity. Typically, ^13^C-flux analysis is used to measure flux through metabolic pathways. ^13^C-flux analysis is amenable to large-scale analysis of mutant collections [8,9]. However, this approach is demanding and cannot be applied as a routine approach to monitor fermentation in real time. Fluorescent indicator proteins (FLIPs) provide a new set of tools for real-time monitoring of metabolite levels in living cells. FLIPs have been applied successfully for monitoring the flux of small molecules in compartments in mammalian cells as well as in intact plant organs [10-16]. The concept makes use of the conformational change of a sensory protein when binding to a ligand (Figure 1). Typically, the FLIP nanosensors consist of a ligand-sensing domain, which is allosterically coupled to a pair of green fluorescent protein (GFP) variants with properties making them suitable for fluorescence resonance energy transfer (FRET). FRET requires donor and acceptor fluorophores with overlapping emission and excitation spectra, respectively. After excitation of the donor, energy is transmitted to the acceptor in a non-radiative manner and emitted by the acceptor. The efficiency of this process depends on the distance between, and relative orientation of, the dipoles of the fluorophores. Ligand-binding-induced conformational changes in the sensors result in altered FRET efficiency, which can be monitored under a fluorescence microscope or in a fluorimeter. The change in FRET can be used as a proxy for levels of the respective metabolites. Periplasmic binding proteins (PBPs) and transcriptional regulators have been exploited successfully for the construction of FLIPs and were used to quantify key metabolites, such as ribose, glucose, maltose, sucrose, glutamate and tryptophan as well as ions such as calcium or phosphate [12,14-19].

To facilitate the development of new nanosensors, here an expression vector is constructed that can be used for sandwiching any ligand-binding domain between FRET-ing GFP variants. In addition, an optimization strategy is applied to improve the signal of the FRET sensor. Given the importance of pentoses such as arabinose and disaccharides such as maltose for fermentation processes in biotechnology, we report the construction of a FRET sensor for the specific intracellular detection of the C5 sugar arabinose and the construction of an optimized nanosensor for monitoring the disaccharide maltose. Most importantly, and as a proof of concept, we demonstrate that these nanosensors can be used to measure steady-state concentrations and to monitor flux in bacteria, specifically *E. coli*, using simple fluorescence spectroscopy, that is, microplate fluorescence readers.

## Results

### Construction of pGWF1, a phage λ recombination vector for terminal fusion of target genes to eCFP and Venus coding sequences

Most FLIP sensors have been constructed by fusion of the N- and C-termini of bacterial PBPs to enhanced cyan fluorescent protein (eCFP) and enhanced yellow fluorescent protein (eYFP) or Venus [4], an improved YFP variant, respectively, using traditional cloning techniques. These fluorescent proteins are variants of the GFP isolated from the marine jellyfish *Aequorea victoria*. To enable the facile construction of FLIPs and streamline the production of novel FLIP sensors, a phage λ recombination vector based on pRSET-B was designed for terminal fusion of target genes to the fluorescent protein variants under control of the T7 promoter. The pRSET-B vector encodes the β-lactamase gene (ampicillin resistance) and the phage f1 origin of replication for site-directed mutagenesis [5]. In addition, the resulting vector pGWF1 sequentially encodes the eCFP gene-*attR1 *site-chloramphenicol acetyltransferase gene-*ccdB *gene-*attR2*-Venus gene. Any domain without a stop codon that has been amplified with primer-encoded attB sites can be sandwiched between eCFP and Venus through pDONR vectors (Invitrogen) using the Gateway technology. The fusion protein carries an N-terminal His_6_-affinity tag for facile purification from *E. coli cell-free extracts*.

### Construction of an arabinose FRET nanosensor

In plants, arabinose is an important constituent of cell wall polysaccharides, for example, the pectic components rhamnogalacturonans and the cellulose-binding glucuronoarabinoxylans. Moreover, arabinose is a major component of hydroxyproline-rich glycoproteins and arabinogalactan proteins. To be able to monitor pentose levels in bacteria, an arabinose FRET sensor was constructed.

**Figure 2:**
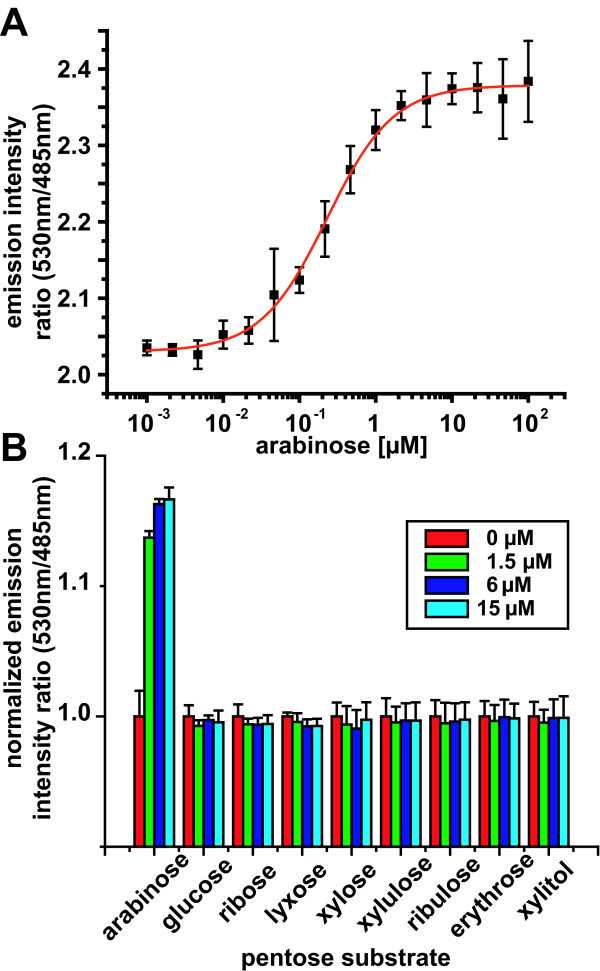
***In vitro *analysis of the arabinose nanosensor FLIPara-200n**. (A) *In vitro *Venus/enhanced yellow fluorescent protein ratio change of affinity-purified FLIPara-250n in the presence of D/L-arabinose. The binding constant *K*_d _of the sensor is 230 ± 18 nM (derived from single-site binding isotherm fit). (B) Substrate specificity of FLIPara-250n as measured by fluorescence resonance energy transfer in the presence of various pentose ligands at three different concentrations. Data were normalized to the sensor in the absence of ligand (first bar on left). Error bars represent the standard deviation (*n *= 3).

The *E. coli *high-affinity L-arabinose binding protein AraF is a polypeptide consisting of 306 amino acid residues that fold in two well-defined lobes connected by a flexible hinge region (Figure 1) [6]. The ligand-binding site is established by the interface of both lobes [6]. A FRET nanosensor for the detection of L-arabinose was constructed by sandwiching *E. coli *K12 *araF *between eCFP and Venus coding sequences of pGWF1. Production of the translated fusion product in *E. coli *was readily detected by recording the emission spectrum of the eCFP-Venus FRET signal in whole cell cultures. When eCFP was excited, significant energy transfer to Venus was detected, resulting in a Venus/eCFP emission ratio of ~2. The addition of D/L-arabinose increased FRET of the purified protein, visible as a decrease in eCFP emission intensity and a concomitant increase in Venus fluorescence intensity, resulting in an increase of about 15% in the Venus/eCFP emission ratio (Figure 2A). The FLIParaF.Ec-250n sensor bound D/L-arabinose with an apparent *K*_d _of 230 ± 18 nM. AraF is stereospecific and binds only L-arabinose. Assuming that the D/L-arabinose mixture consists of equal parts of D and L isomer, the *K*_d _of the arabinose FRET nanosensor for L-arabinose would be 115 nM, which is similar to that of unmodified AraF as measured by tryptophan fluorescence spectroscopy (98 nM) [7]. The fusion protein, denoted by FLIParaF.Ec-250n, specifically interacts with arabinose, as several other pentose substrates did not induce a ratio change (Figure 2B).

### Monitoring intracellular arabinose levels in *E. coli*

**Figure 3:**
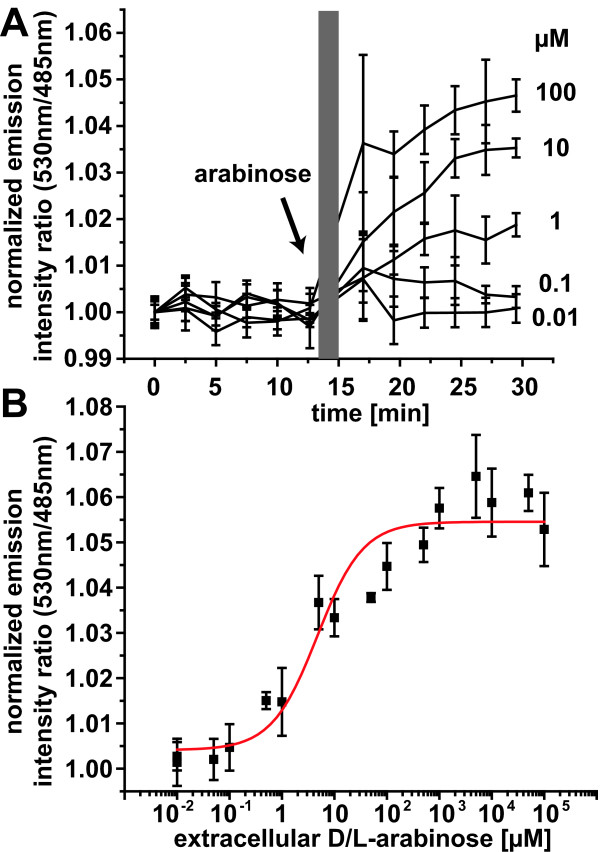
**Detection of intracellular arabinose with FLIPara-200n**. (A) Detection of intracellular arabinose in *Escherichia coli *BL21-Gold(DE3) cells expressing FLIPara-250n. The arrow indicates arabinose addition (note that here the addition was performed by hand, leading to an interruption of the protocol, thus rates cannot be deduced; marked as a gray bar). Error bars indicate the standard deviation of at least three replicates. (B) Arabinose-dependent sensor *in vivo *response at *t *= 25 minutes. The intracellular arabinose concentration that corresponds to the affinity constant (*K*_d_) of FLIParaF.Ec-250n is reached at an external arabinose concentration of 4.7 ± 2.0 μM, indicating that the intracellular concentration is around 20-fold lower compared with the extracellular concentration in a medium containing 4.7 mM arabinose. Error bars represent the standard deviation (*n *= 3).

The FLIParaF.Ec-250n nanosensor was expressed in *E. coli *BL21-Gold(DE3). Arabinose was added at concentrations between 0.01 and 100 μM to the *E. coli *cell cultures in microtiter wells, and arabinose levels were monitored by recording the Venus/eCFP emission ratio derived from FLIParaF.Ec-250n (Figure 3A). Initial monitoring in the absence of arabinose gave a stable baseline. The emission ratio did not change until after the addition of at least 0.1 μM arabinose and increased between 0.1 and 100 μM of external arabinose. Rates could not be determined in this case since the data acquisition was interrupted for the manual addition of arabinose. Cytosolic arabinose levels saturated above 500 μM to 10 mM (Figure 3B), indicating that the sensor accurately reports changes in intracellular arabinose concentration. Plotting of the emission ratios against the external arabinose concentration yields an *in vivo *response curve which indicates that the apparent steady-state levels of arabinose are about 20-fold lower compared with the external supply (under the assumption that the affinity of the sensor is not affected in the cytosolic environment; Figure 3B). The signal-to-noise ratio of the *in vivo *analysis was relatively high; however, an improvement of the signal by linker deletions or fluorophore insertion as successfully achieved for the glucose and glutamates sensors [8] should provide a means to obtain reliable *in vivo *quantification.

### Construction of an improved maltose sensor

**Figure 4:**
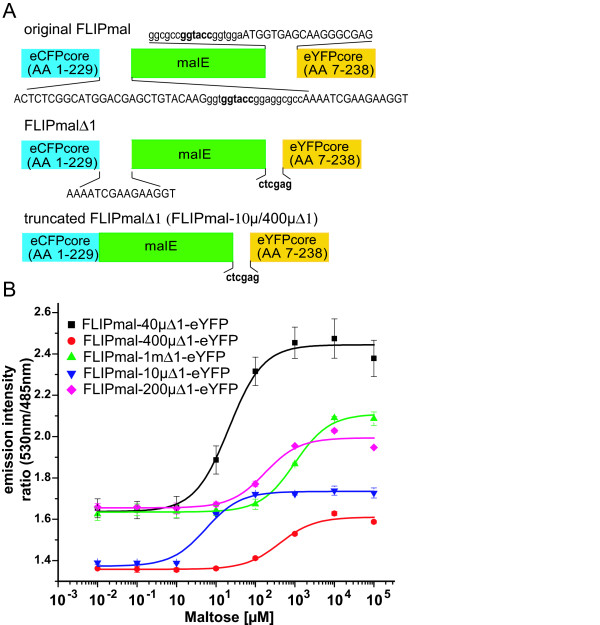
**Construction and *in vitro *analysis of improved maltose sensors**. (A) Sketches of the original and improved versions of the FLIPmal sensors with shortened linkers. The amino acid sequence in capital letters corresponds to enhanced cyan fluorescent protein/enhanced yellow fluorescent protein or malE; small letters correspond to the synthetic linkers (sequence in bold is the restriction site). (B) *In vitro *fluorescence resonance energy transfer ratio changes of the FLIPmal sensor variants in the presence of maltose. Error bars represent the standard deviation (*n *= 3).

To test whether this simple detection system can be used for monitoring cytosolic maltose levels, and to test whether an increased sensitivity of the assay can be obtained with optimized FRET sensors, a set of improved maltose sensors was constructed by linker deletions and expressed in *E. coli*. A maltose sensor FLIPmal-25μ had previously been constructed by fusing the *E. coli *MalE protein with eCFP and eYFP fluorescent proteins [9]. However, FLIPmal-25μ showed only a modest ratio change in the presence of maltose. Linker deletions have been used successfully to improve the ratio change of glucose FRET sensors [8], and the same strategy was applied here to the maltose sensor, resulting in a set of maltose sensors with *K*_d_s ranging from the low to high micromolar range (Figure 4). FLIPmal-40μΔ1-eYFP showed a four-fold increase in eYFP/eCFP ratio change compared with the original long linker construct FLIPmal-25μ.

In addition, a sensor was constructed in which the FRET acceptor fluorophore eYFP was replaced with the eYFP variant Venus. Compared with eYFP, Venus has a reduced maturation time, and is brighter and less sensitive to environmental factors [4], potentially resulting in a more robust maltose nanosensor. FLIPmal40μΔ1-V is characterized by an apparent *K*_d _of 37 μM and exhibited no apparent differences in its ligand-binding properties compared with the variant with eYFP (data not shown).

### Monitoring maltose flux in *E. coli*

**Figure 5:**
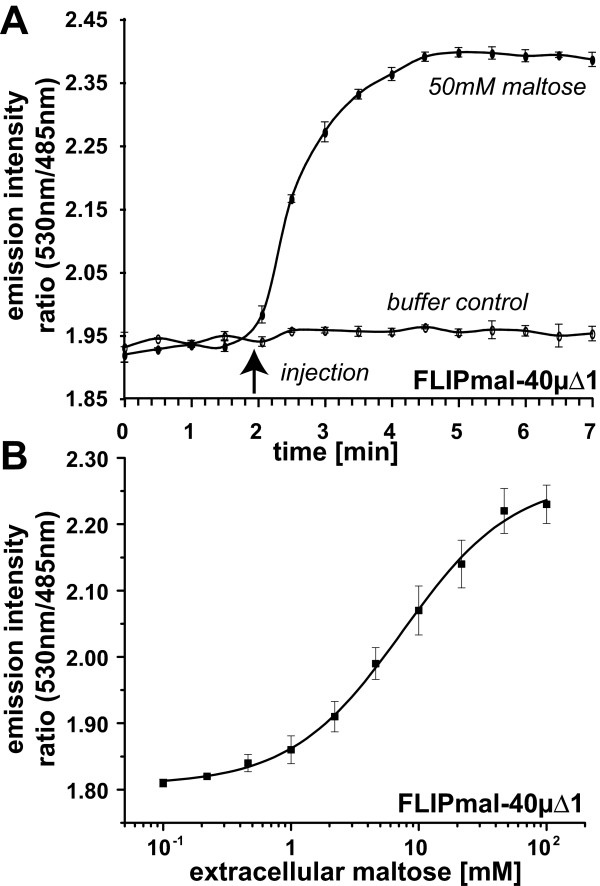
**Accumulation of intracellular maltose detected with FLIPmal sensors**. (A) Cytosolic maltose accumulation measured in 30-second intervals after injection of buffer or 50 mM maltose to microtiter plate wells containing *Escherichia coli *BL21-Gold(DE3) cells expressing FLIPmal-40μΔ1-enhanced yellow fluorescent protein. The arrow indicates the time point of maltose addition using the injector of the Tecan Infinite M200 fluorimeter (note, in contrast to Figure 3A, this procedure allows the determination of rates). (B) Dose-response curve for maltose detected by FLIPmal-40μΔ-enhanced yellow fluorescent protein in *E. coli *BL21-Gold(DE3) cells. Intracellular maltose levels are around 700-fold lower compared with the external concentration (*K*_0.5 _= 7.4 mM). Error bars represent the standard deviation (*n *= 3).

FLIPmal40 μΔ1-eYFP was expressed in *E. coli *BL21-Gold(DE3) and cells were transferred to 96-well microplates. To be able to follow the rate of maltose accumulation in *E. coli*, maltose was injected directly into the wells containing the bacterial cells in a microplate fluorimeter equipped with injectors. Either buffer or 50 mM maltose was injected and the eYFP/eCFP ratio was monitored in 30-second intervals (Figure 5A). While the injection of buffer had no effect on the relative intensities of eYFP and eCFP, the addition of 50 mM maltose led to a time-dependent accumulation of maltose, which reached a maximum within 2.5 minutes. The *in vivo *FRET ratio change of FLIPmal-40μΔ1-eYFP was more than three-fold higher compared with that of FLIPmal-25μ (data not shown), and provided a high signal-to-noise ratio, demonstrating the suitability of the linker variant sensor for the quantitative analysis of cytosolic maltose levels as well as maltose flux rates, which are composed of the rate of influx minus the rate of metabolism.

To determine the steady-state levels of maltose at different maltose concentrations, maltose was added to *E. coli *cells expressing FLIPmal-40μΔ1-eYFP and eYFP/eCFP ratio changes were monitored (Figure 5B). Initial monitoring of the emission ratios yielded a stable baseline. Upon the addition of maltose, the ratio increased in a concentration-dependent manner. When the sensor response (after reaching a steady state) was plotted against the external maltose concentration, the concentration-dependent *in vivo *response can be fitted with a single-site binding curve. The apparent *in vivo K*_0.5 _was 7.4 mM; thus, the apparent intracellular steady-state maltose level is about 180-fold lower relative to external supply.

## Conclusion

FLIP sensors have been developed for a wide range of ions, amino acids and mono- and disaccharides and have been used to address important biological problems, such as the release of glutamate from neurons [10,11], the exchange of tryptophan against kynurenine in cancer cells [12] or the transport of glucose across the endoplasmic reticulum membrane [13]. Here it has been shown that the nanosensor technology also has potential applications for fermentation processes in the food, pharmaceutical and biofuel industries, since the FRET sensors robustly and quantitatively report changes in intracellular ligand concentrations in microorganisms. In this study, both a novel arabinose FRET nanosensor as well as an improved maltose nanosensor were used to quantify intracellular ligand concentrations in bacterial cell cultures in a 96-well microplate fluorimeter. These results demonstrate that, after addition of sugar, the rate of uptake exceeds the rate of metabolism in *E. coli*. Moreover, by using an injection module attached to the fluorimeter, it was possible to determine accumulation rates after the addition of the metabolite. Finally, the new Gateway-based vector system used here to construct the arabinose sensor will help to accelerate the development of novel sensors for other relevant compounds including xylose, one of the major cell wall-derived pentoses.

The apparent cytosolic steady-state levels of both arabinose and maltose were 20- to 200-fold lower in the cytosol compared with the extracellular medium, indicating the presence of a highly active metabolic flux that exceeds uptake rates even at high external levels. It should be noted that the maltose sensor also recognizes maltooligosaccharides [9]; thus, the actual maltose levels in the cytosol may be even lower if maltose is rapidly converted into maltooligosaccharides. At low maltose concentrations, maltose is imported by the maltoporin LamB across the outer membrane, and then bound by the maltose PBP, which delivers maltose to the ATP-dependent ABC transporter [14]. However, at high maltose levels, this system with an affinity of around 1 μM (LamB) is not essential since the growth of *lamB *mutants is unaffected at high maltose concentrations [15]. Consistent with the responses observed here, low-affinity/high-capacity uptake systems predominate at 1 to 100 mM maltose (stationary BL21-Gold(DE3) cells, kept in LB at 4°C overnight to allow full folding of the fluorophores, then washed and incubated in M9 medium for 1 hour to ensure low endogenous maltose levels). Given the successful use of the sensors in other systems, it will be possible to use physiological conditions; moreover, this system can be used to identify the nature of the low-affinity transport system.

The carbohydrate polymer loading in lignocellulosic biomass feedstock saccharification is typically at least 10%, resulting in monosaccharide concentrations up to several 100 mM, which is higher than encountered by fermentative organisms in nature and might result in increased carbon flux into undesired pathways [16]. In addition, different organisms have a preference for fermenting one monosaccharide over another resulting in inefficient use of the feedstock substrate [17]. Nanosensors might aid in identifying conditions of metabolite accumulation as a result of obstruction in, or repression of, metabolic pathways. An absence of, or decrease in, intracellular metabolite levels would indicate a lack of external supplies caused for instance, by the inhibition of uptake systems.

In this study, a sensor for monitoring intracellular levels of the pentose arabinose has been described, which was constructed by sandwiching the arabinose-binding protein gene of *E. coli *between the fluorophore genes in the Gateway destination vector pGWF1. The other main pentose present in cell walls of biofuel feedstocks such as corn fiber is xylose [18]. Given the success in generating FRET sensors for ribose, arabinose, glucose, maltose and sucrose using this concept [9,19-21], analogously, xylose sensors may be constructed using identified xylose-binding proteins as sensing domains [22,23]. The developed Gateway-based cloning system enables rapid fusion of new recognition elements into a vector containing flanking fluorescent proteins. In addition, the linker deletion strategy used for optimization of the maltose sensor can be applied to metabolite sensors with high signal-to-noise ratios such as those shown here for the arabinose and unmodified maltose sensors. Given the wide spectrum of naturally available scaffolds that can be used for constructing additional FRET sensors relevant for metabolite analysis in relation to biofuels, the Gateway system and linker optimization strategy promise the rapid development of a suite of efficient FRET sensors for applications in bioengineering.

Furthermore, the high dynamic range of the responses, specifically shown in Figure 5A for maltose accumulation in *E. coli *make them suitable for use in high-throughput screens in, for instance, strain optimization. The technology is simple and fast to use and complements flux analysis by alternative methods, such as isotope pulse labeling combined with mass-spectroscopy [24]. The data shown here demonstrate that FRET nanosensors can be used in prokaryotes and as such, have potential applications for the development of strains for the production of biofuels. The analysis of metabolite flux using an array of sensors will be valuable for ongoing efforts in kinetic modeling of carbohydrate fermentation in biofuel-producing organisms such as *Zymomonas mobilis *[25].

## Methods

### Chemicals, strains and plasmids

All chemicals, including D/L-arabinose and maltose were of analytical grade and purchased from Sigma-Aldrich. *E. coli *TOP10F' was used as the cloning host and *E. coli *BL21-Gold(DE3) was used as the protein production host. pRSET-B (Invitrogen) and pGWF1 (see below) were used for *E. coli *expression. eCFP and eYFP were obtained from Clontech; Venus was a generous gift from Dr Atsushi Miyawaki, Riken, Japan.

### Construction of pGWF1, a Gateway vector for linear fusion of target genes between eCFP and Venus coding sequences

The gateway-compatible expression vector pGWF1 was constructed from pFLIPmal (malE_Ec with N-terminal eCFP and C-terminal eYFP; see [9]) first by constructing pFLIPmal_Venus via cloning of a Venus polymerase chain reaction (PCR) product into the *AgeI *and *HindIII *sites, replacing eYFP with Venus. Subsequently, unique *XhoI *and *KpnI-EcoRV *sites were introduced at the 5'- and 3'-ends of the *eCFP *coding region by site-directed mutagenesis; *EcoRV*-*SpeI *and *PspOMI *sites were introduced at the 5'- and 3'-ends of the *Venus *coding region. This construct was digested with EcoRV, producing a blunt-ended vector into which the Gateway Reading Frame A (a 1.6-kb fragment containing the chloramphenicol-resistant gene and the *ccdB *gene flanked by *attR *sites) was cloned according to manufacturer's protocol (Invitrogen).

### Construction of an arabinose FRET nanosensor

The *araF *gene (EMBL: K00420) was amplified from *E. coli *K12 genomic DNA using homologous primers (p1: ggggacaagtttgtacaaaaaagcaggctcgggtactcattcgtttttgccctacacaaaa, p2: ggggaccactttgtacaagaaagctgggttactagtcttaccacctaaaccttttttctcc (forward and reverse, respectively, *araF *sequence underlined), resulting in a PCR product that consisted of *araF *without a stop codon flanked by phage λ *attB *recombination sites. The *araF *gene was introduced into pDONR (Invitrogen) using a base pair recombinase reaction (Invitrogen) and was mobilized into pGWF1 (in frame fusion between eCFP and Venus coding sequences) using a LR recombination reaction (Invitrogen). The final construct was denoted by pGW1araF.Ec and the correct sequence of the *araF *gene was verified by DNA sequencing (Sequetech). The gene product encoded on pGW1araF.Ec was denoted by FLIParaF.Ec-250n.

### *In vitro *characterization of the arabinose FRET nanosensor

FLIP constructs were transferred to *E. coli *BL21-Gold(DE3) and nanosensor proteins were produced and purified as described in [9]. Purified sensor protein was added to a dilution series of ligand in 20 mM 3-(N-morpholino)propanesulfonic acid pH 7.0 in the range of 10^-4 ^to 10^-9 ^M and analyzed in a monochromator microplate reader (Safire, Tecan, Austria; eCFP excitation at 433 nm with 12 nm bandwidth, eCFP emission monitored at 485 nm with 12 nm bandwidth and Venus emission monitored at 530 nm with 12 nm bandwidth). eCFP emission is characterized by two emission peaks at 476 and 501 nm [26]. The eCFP emission used for the ratio calculation was determined at 485 nm. Protein was diluted to give relative fluorescence units (RFUs) for Venus and eYFP of 20,000 to 30,000 RFU at a gain of 70 to 75. By using the change in relative emission intensities at 530/480 nm upon binding of ligand, affinity constants (*K*_d_) were determined by fitting the titration curves to a single-site-binding isotherm:

*R *= *R*_apo _+ (*R*_sat _- *R*_apo_)·(*n *· [*L*])/(*K*_d _+ [*L*])

with: [*L*], ligand concentration; *n*, number of equal binding sites; *R*, ratio; *R*_apo_, ratio in the absence of ligand; *R*_sat_, ratio at saturation with ligand. Three independent protein preparations were analyzed; each protein preparation was analyzed in triplicate.

### Construction of improved maltose FRET nanosensors

FLIPmal-225 μ, carrying the mutation W62A, was used as a base for generating improved FLIPmal sensors [9]. Seventy-five nucleotides (25 amino acids; Figure 4A) of the linker regions were removed in FLIPmal-225 μ by site-directed mutagenesis [5] similar as previously done in the FLIPgluΔ13 series [8]. This new FLIPmal sensor had a *K*_d _for maltose of 200 μM and was denoted by FLIPmal-200 μΔ1-eYFP (FLIPmal-200 μΔ1-eYFP carries the mutation W62A). Affinity mutants of this sensor were created by structure-guided, site-directed mutagenesis of the binding pocket, yielding FLIPmal-40μΔ1-eYFP (carrying the mutation W230A in addition to W62A) and FLIPmal-1mΔ1-eYFP (carrying the mutation Y155A in addition to W62A). In addition, two sensors were constructed in which the first five amino acids of malE were deleted, which earlier had been shown to generate a sensor with a *K*_d _in the low micromolar range [9]. This five-amino-acid deletion in the W62A background gave a sensor with a *K*_d _of 400 μM (FLIPmal-400μΔ1-eYFP). When W62A was reverted back to W62W, the affinity of the sensor was found to be 10 μM (FLIPmal-10 μΔ1-eYFP). When the clones were sequenced, an additional mutation (N227A) was found in FLIPmal-40μΔ1-eYFP, which probably occurred as an artifact during site-directed mutagenesis.

### Monitoring of accumulation and relative flux rates using the FRET nanosensors

*E. coli *BL21-Gold(DE3) cells were transformed with pGW1FaraF.Ec, p982 or p3367, which encode an arabinose FLIP nanosensor with a *K*_d _of 200 nM, a maltose FLIP nanosensor with a *K*_d _of 25 μM, and a maltose FLIP nanosensor with a *K*_d _of 40 μM, respectively. Cultures were incubated in LB in baffled Erlenmeyer flasks for 48 hours in the dark at room temperature while shaking, and stored for 16 hours at 4°C to ensure appropriate folding of the fluorophores. Cultures, 5 ml, were sedimented, washed in 10 ml M9 minimal salts medium, sedimented and resuspended in 9 ml M9 medium. Cells were dispended in a microplate at 90 μl per well. The sensor output was monitored in a microplate fluorescence spectrophotometer with indicated intervals. After a stable baseline was obtained, 10 μl of various concentrations of arabinose or maltose in M9 medium were added to the cells and the fluorophore emissions were recorded for another 20 minutes. eCFP ratios were normalized against cells to which 10 μl M9 medium had been added. To monitor accumulation rates, the injection module of a Tecan Infinite M200 (Tecan, Austria) was used.

## Abbreviations

eCFP, enhanced cyan fluorescent protein; eYFP, enhanced yellow fluorescent protein; FLIP, fluorescent indicator protein; FRET, fluorescence resonance energy transfer; GFP, green fluorescent protein; PBP, periplasmic binding proteins; PCR, polymerase chain reaction; RFU, relative fluorescence unit.

## Competing interests

The authors declare that they have no competing interests.

## Authors' contributions

DC carried out *in vitro *tests and optimized the *in vivo *tests. TK planned and developed the *in vivo *tests and wrote parts of manuscript. LLL and DC constructed the arabinose sensors. IL planned and supervised the linker deletions of the maltose sensor and carried out the analysis of the *in vitro *response. WBF conceived of and planned the overall work, and wrote parts of the manuscript.
